# One digital health through wearables: a viewpoint on human–pet integration towards Healthcare 5.0

**DOI:** 10.3389/fdgth.2025.1668739

**Published:** 2025-12-18

**Authors:** Mostafa Haghi, Samira Abani, Soheil Khooyooz, Anice Jahanjoo, Samaneh Rashidibajgan, Nima TaheriNejad, Thomas M. Deserno, Holger Volk

**Affiliations:** 1 ECLECTX Team, Institute for Computer Engineering, Heidelberg University, Heidelberg, Germany; 2 Department of Small Animal Medicine and Surgery, University of Veterinary Medicine Hannover, Hannover, Germany; 3 Centre for Systems Neuroscience, University of Veterinary Medicine Hannover, Hannover, Germany; 4 Institute of Computer Technology, TU Wien, Vienna, Austria; 5 School of Computer Science and Engineering, Constructor University, Bremen, Germany; 6 Peter L. Reichertz Institute for Medical Informatics of TU Braunschweig and Hannover Medical School, Braunschweig, Germany

**Keywords:** digital technology, Healthcare 5.0, human health, one digital health, one health, pet health, wearable devices

## Abstract

Wearable technologies mark a transition in healthcare evolution, from paternalistic (Healthcare 1.0) to reactive (Healthcare 2.0), proactive (Healthcare 3.0), and data-integrated care (Healthcare 4.0). The next stage, Healthcare 5.0, envisions the seamless integration of human and pet health data, fostering a more holistic approach to disease prevention and management. In this viewpoint, we explore the disruptive potential of integrating health monitoring between humans and pets through wearable technology, highlighting the interconnected nature of human-pet health. We examine the parallel evolution of human and pet health monitoring, assessing current technologies and their potential to enhance both fields. We discuss that wearable technologies not only improve chronic disease management but also enable early detection of zoonotic and emerging diseases. Additionally, we emphasize the potential re-usability of human wearable devices for pets, outlining the associated technical challenges. This can lower costs and accelerate adoption, offering mutual benefits for both domains. We address the need for an integrated, linked platform that enables real-time data analysis. Data integration ultimately results in better diagnostic accuracy, optimized treatment plans, and enhanced quality of life for humans and pets. Re-purposing wearables for human-pet health monitoring enables real-time data collection, predictive analytics, and prevention to accelerate the implementation of Healthcare 5.0.

## 1 Introduction

### 1.1 Health holistic approaches

Humans and their furry companions share a unique bond and encounter a range of similar health concerns. Despite thousands of households worldwide welcoming pets, there is still an absence of integrated health monitoring systems jointly addressing the well-being of both [1]. Quality of life (QoL), both physical and mental, as well as perspectives on health, well-being, safeguarding, and environmental factors, require holistic and interdisciplinary approaches. In this context, QoL as defined by the WHOQOL framework, encompasses physical, psychological, social, and environmental dimensions [2]. Wearables and connected technologies extend QoL assessment beyond episodic encounters to continuous, context-aware monitoring of sleep, mobility, and participation. In human-companion-animal ecosystems, shared environments and daily routines link animal welfare directly to human QoL, enabling earlier detection of decline and more targeted interventions. These approaches emphasize the interconnected relationship between humans, animals, and the environment [3]. Three influential concepts of One Health, EcoHealth, and Planetary Health, illustrate this linkage. Although they share foundational elements and conceptual similarities, they differ in their focus and scope [4]. One Health aims to improve well-being by preventing risks and mitigating crises at the human-animal-environment interface and emphasizes a whole-of-society approach [5, 6]. EcoHealth extends this perspective by explicitly integrating environmental sustainability and socioeconomic stability [7]. Planetary Health, while less interdisciplinary, concentrates primarily on human health within environmental boundaries [4]. Despite these differences, all frameworks converge on a central message: human, animal, and environmental health are inseparable, and technological advances must reflect this interconnectedness to achieve meaningful impact.

### 1.2 Bridging the paradigms and technologies: one digital health

In 2018, the World Health Organization (WHO) released a comprehensive taxonomy of Digital Health, identifying numerous aspects of this rapidly expanding field [8]. Investments in Digital Health are substantial, for example, reaching $6 billion in 2017 [9], reflecting its potential to enhance healthcare delivery and support public health systems [10]. Digital Health interventions increasingly benefit humans, animals, and ecosystems [11] by leveraging two primary dimensions: (i) real-time big data streams and (ii) wearable and tracking technologies that strengthen disease surveillance, early-warning systems, preparedness, and response by integrating traditional surveillance with new data sources [12].

**Figure 1:**
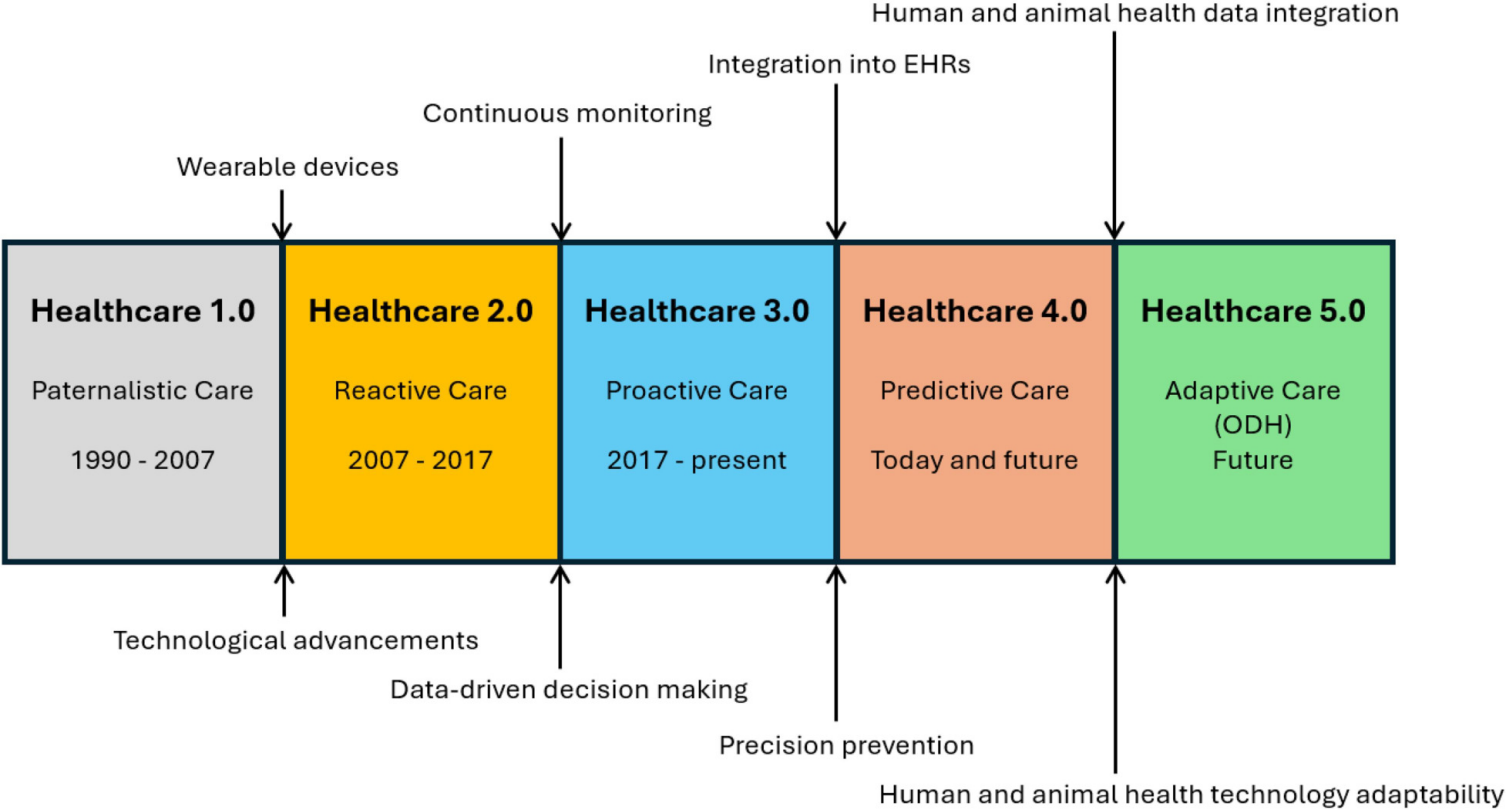
Wearable technology is a key in Healthcare evolution, enabling continuous monitoring, data integration into EHRs, and fostering the convergence of human and animal health data. Healthcare 5.0 yields adaptive care across species.

The One Digital Health (ODH) concept bridges the gap between holistic health approaches and technological advancements [13]. ODH defines the components of the digital health ecosystem and explores how emerging technologies can support healthcare delivery and promote overall well-being across species. A central aspect of ODH is the integration of human and veterinary medical data into real-time information systems, an essential step in advancing public health, particularly where human and animal environments intersect. Historically, healthcare was delivered in a largely paternalistic manner (Healthcare 1.0); With the implementation of wearable devices, the paradigm shifted to reactive care (Healthcare 2.0); Continuous monitoring enabled proactive care (Healthcare 3.0), and is already pushing the field toward predictive care (Healthcare 4.0); By the integration of human and animal health data, we will approach Healthcare 5.0 (Figure 1) [14, 15].

In this viewpoint, we examine the role of wearable technologies across five key areas of (i) management of chronic conditions, (ii) monitoring physical activity, (iii) behavioral and mental health, (iv) early disease detection, and (v) clinical applications. We emphasize ODH contributions to the health of humans and companion animals (specifically dogs and cats) and highlight how interconnected data streams reveal cross-species insights, such as identifying common stressors. We also discuss technical challenges and opportunities in adapting human wearables for animal use. Finally, we outline a roadmap for advancing integrated, cross-species wearable health monitoring systems.

## 2 Human and pet health: a parallel journey

### 2.1 Human-pet interaction, health, and its implications

In 2024, 46% of German households (18.45 million homes) host a pet [16], most commonly dogs and cats, followed by small mammals, such as rabbits and guinea pigs, birds, fish, and reptiles [16]. The German pet market increased by €4 billion in two decades to €7.1 billion in 2023 [16]. Pet owners increasingly consider their pets as integral family members [17], showing the importance of pet health, which directly extends to the QoL of owners and the broader community [18, 19], particularly when managing chronically ill pets with conditions like epilepsy, diabetes, allergy, cardiovascular diseases, dementia, and cancer [20]. Pets in poor health often trigger a domino effect, ranging from rising pet health insurance premiums to affecting the well-being of pet owners. Neurological conditions such as epileptic seizures particularly strain owners emotionally, not only because of their episodic nature but also due to medication side effects and clinical signs including ataxia, sedation, and behavioral changes [21, 22].

### 2.2 Shared metrics and benefits

“We are more alike than we might think.”

#### 2.2.1 Overlapping health parameters

The parallels between humans and animals extend far beyond emotional connection and companionship. Companion animals, much like humans, face many of the same environmental stressors, including air pollution and noise, along with socioeconomic influences contributing to their health and well-being [23]. Whether engaging in the same physical activity or exchanging countless gut microbes, companion animals accurately reflect human well-being [24]. These shared physiological markers are detected through different sensing modalities and mechanisms such as Photoplethysmography (PPG), Electrocardiography (ECG) derive heart rate and heart rate variability (HRV) by measuring changes in blood volume or electrical cardiac activity; acoustic sensors capture subtle mechanical vibrations associated with pulse and breathing; accelerometers detect movement, gait, posture, and sleep–wake cycles through motion signatures; and temperature sensors measure peripheral thermoregulation and fever-related changes. Because the same physical principles apply across species, the resulting signals are directly comparable, enabling unified interpretation of stress, activity, rest quality, and early deviations from baseline in both humans and companion animals. This creates a comparable dataset that allows shared stressors or early signs of decline to be detected objectively across humans and pets.

Over the past few years, smart collars for dogs and cats have gained popularity as they enable remote, non-invasive, and continuous monitoring of vital signs. Latest devices integrate thermometers for measuring temperature, acoustic sensors for pulse, HRV, and respiratory acquisition, accelerometers for activity, calories, and posture detection, and global positioning systems (GPSs) for location and tracking [25]. These devices track various behaviors, including scratching, licking, sleeping, eating, drinking, running, walking, and resting. Analyzing such data provides insights into a pet’s overall health. For instance, increased licking or scratching suggests allergies or dermatological conditions [26], while reduced activity or restlessness indicates discomfort [27] from conditions such as osteoarthritis, cardiovascular disease, or neurological disorders [28]. Abnormal eating or drinking patterns indicate metabolic disorders, such as feline hyperthyroidism or canine hyper-adrenocorticism [29]. Changed walking or running patterns indicate pain, fatigue, epilepsy, or feline urethral obstruction [30]. By quantifying these behaviors through sensors, wearables translate clinical or subclinical signs into continuous digital biomarkers, enabling earlier intervention and facilitating cross-species comparison of lifestyle, stress, and disease patterns.

#### 2.2.2 Common diseases between humans and pets

Owning a companion animal contributes to various health benefits, including regular physical activity and enhanced mental well-being [31]. However, it is crucial to acknowledge the potential downsides, including numerous zoonotic pathogens that companion animals transmit to humans, directly or indirectly. For instance, soil-transmitted helminth infections by various species of parasitic worms are among the most common worldwide [32]. Dogs and cats play a major role in contaminating the environment and potentially serve as reservoirs for infections in humans. Additionally, a fatal viral disease like rabies can be transmitted through a bite, mainly by domestic dogs [33]. Cats are carriers of toxoplasmosis, caused by the single-celled parasite Toxoplasma gondii [34]. Cystic echinococcosis is another severe, occasionally fatal parasitic disease caused by the larval cystic stage of the dog tapeworm Echinococcus granulosus [35]. In addition to zoonotic diseases (animal-to-human), we need to consider reverse zoonosis, causing (human-to-animal) harm. Examples are influenza, SARS-CoV-2, and Monkeypox viruses [36]. Wearables cannot detect pathogens directly, but by monitoring early physiological deviations, such as fever, reduced mobility, abnormal respiration, or sleep disruption, they provide an important layer of early-warning signals in both humans and pets, complementing traditional public-health surveillance systems.

## 3 Integration of health monitoring

### 3.1 Wearable devices for connected health

Connected Health is a patient-centred model that encompasses wireless, digital, electronic, mobile, and tele-health solutions, in which devices, services, or interventions are designed around patients’ needs and health data are shared to enable proactive and efficient care [37]. Within this paradigm, telemedicine is a core component that enables quicker and more efficient diagnosis and clinical care by bridging physical distances and extending spatial-temporal monitoring [38]. Wearable technologies such as fitness trackers, smart watches, wearable ECG recorders, and smart clothing facilitate real-time, non-invasive monitoring of health parameters (e.g., vital signs) [39, 40]. Offering real-time feedback, wearables are an objective tool and beneficial for the elderly, rehabilitation, and those with disabilities. Furthermore, wearables facilitate home-based rehabilitation and save over $200 billion in healthcare costs by reducing clinician-patient interaction time as well as transportation efforts [41].

Smart devices help managing sleep [42], stress [43], productivity, and chronic diseases [44], detect depression, and monitor activities [45]. Wearables have applications in stroke recovery [46], assessing muscle activity [47], and bear the potential to replace magnetic resonance imaging (MRI) for brain activity monitoring [48]. In chronic conditions like chronic obstructive pulmonary disease (COPD), wireless pulse oximeters enhance self-management and reduce hospital admissions. Apple’s ECG has received Food and Drug Administration (FDA) and European approval, indicating wearables’ growing importance [49]. During the COVID-19 pandemic, wearables aided in remote monitoring and consultations for chronic and post-COVID conditions [50].

### 3.2 Wearable devices for pets

**Figure 2:**
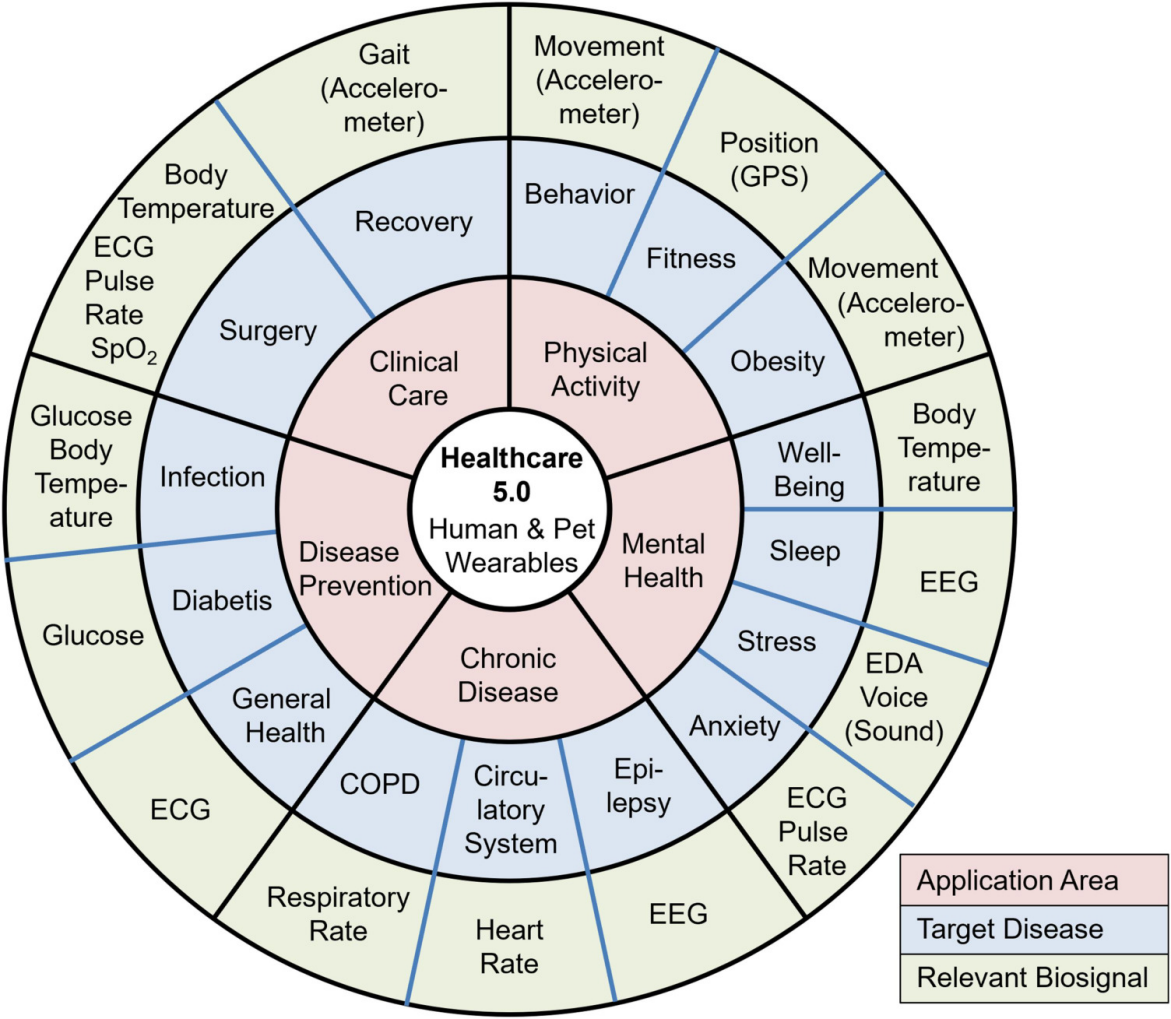
Healthcare 5.0 supported by wearables. Red: main application area; Blue: target disease; Green: relevant biosignal. Application-centered map of reusable human ↔ pet wearables across five primary domains: clinical care (post-op recovery, vital-sign surveillance), physical activity (activity profiling, gait), disease prevention (early anomaly detection, exposure/risk monitoring), chronic disease (cardiometabolic and respiratory follow-up), and mental health (stress, affective state). Each application links to representative conditions/indicators (e.g., respiratory dysfunction, mobility/ataxia, insomnia, anxiety) and their enabling biosignals/modalities such as ECG, IMU/accelerometry, EEG, EDA, and skin temperature. This depicts interoperability and bidirectional reusability of sensing and data across species within telemedicine-like connected-health pathways toward Healthcare 5.0.

The global market in wearables for pets was $2.70 billion in 2023 and is expected to grow by 14.3% from 2024 to 2030 [51]. Wearable devices for companion animals increasingly enable continuous and remote health monitoring, conceptually analogous to human Remote Patient Monitoring Systems (RPMS), although the terminology is not yet standardized in veterinary medicine [52, 53]. Figure 2 provides an overview of the cross-species mapping between biosignals, representative conditions, and major applications. It visualizes the cross-species health ecosystem enabled by wearable technologies through mapping three interconnected layers: application areas (inner ring), target diseases or conditions (middle ring), and the biosignals required to monitor them (outer ring). This structure shows the direct linkage between measurable physiological markers such as heart rate, respiratory rate, glucose, sleep, or movement and specific clinical or behavioral conditions relevant to both humans and companion animals. By organizing these elements around the Healthcare 5.0 framework, the figure illustrates how shared biosignals enable unified approaches to chronic disease management, mental health assessment, physical activity monitoring, clinical care, and disease prevention across species. One of the primary applications is monitoring physical activity. Equipped with accelerometers and GPS, pet devices provide detailed data to support the management of obesity and ensure sufficient exercise [54]. Deviations from normal activity patterns signal underlying health issues. According to Chambers et al., wearable accelerometers accurately quantify the intensity and duration of physical activity in dogs, thereby aiding in weight management and maintaining physical fitness [55]. Wearable technologies further promise to detect and manage chronic conditions in pets. In particular, their applications for monitoring canine epilepsy collect objective data on seizure frequency and duration, which supports more informed and individualized treatment planning [56]. Similarly, Oliveira et al. employ wearable pulse oximeters and heart rate monitors to manage respiratory and cardiovascular conditions [57].

Another application is in the early detection of diseases and health anomalies. Brugarolas et al. explore the use of wearable biosensors in detecting physiological changes correlated to stress, allowing for earlier interventions [58]. Foster et al. demonstrate the feasibility of reconstructing ECG, heart rate, respiration rate, and body movement of dogs during rest and sleep [59]. Moreover, behavioral monitoring using wearable devices detects anxiety, enabling timely behavioral interventions [60]. Similarly, tracking sleep patterns and restlessness in pets provides insights into their mental health and overall well-being [61].

Wearables also support the management of specific health conditions. For example, in diabetic pets, continuous glucose monitoring through wearable devices enables better disease control [62]. These devices assist veterinary surgeons in tracking disease progression and assessing therapeutic outcomes. Engelsman et al. demonstrate effective gait analysis to quantify ataxia in dogs [63].

### 3.3 Recognizing the disconnect in human and pet health monitoring

#### 3.3.1 Major challenges

**Technology and tools:** Technological progress in health monitoring differs between humans and pets. Humans have access to sophisticated, high-performance devices, whereas pet health monitoring remains more limited and often passive [64]. Although pets benefit from the evolution from Healthcare 1.0 to 4.0 such as wearables, telemedicine, data-driven care, platform integration, and preventive approaches [65], the veterinary domain still does not realize the full potential of Healthcare 4.0.
**Data availability and integration:** Human healthcare emphasizes continuous data collection, multimodal analysis, and integration of diverse data streams for preventive care and early disease detection. For example, Accident and Emergency Informatics (A&EI) uses the International Standard Accident Number (ISAN) to securely exchange private data across silos [66]. In contrast, pet health relies largely on intermittent evaluations at veterinary visits, and information from different sources including veterinary records, home monitoring, and dietary data, remains fragmented [67].
**Accessibility and affordability:** While advanced technologies for humans are widely available and relatively cost-effective, equivalent pet technologies are less accessible and often more expensive, limiting adoption among pet owners.

#### 3.3.2 Boosting factors

**Lack of data protection for pets:** The implementation of robust data protection frameworks such as the General Data Protection Regulation (GDPR) challenge researchers in sharing and utilizing personal health data [68]. While these measures are important for protecting personal data, as enshrined in the EU Charter of Fundamental Rights, there are no specific data protection rights for animals. We see this gap as an opportunity for researchers to boost veterinary medicine and Healthcare 5.0.

## 4 Perspective and roadmap

### 4.1 Connected human and pet health

#### 4.1.1 Human wearables for pets

Although the anatomy of humans and animals differs, human wearables are repurposed for pets. This is an initial step to overcome current limitations, such as passive monitoring, availability, and affordability. A major challenge is adapting size, shape, and fit to different anatomies, ensuring comfort, safety, and irritation-free use [69]. Devices must be durable, non-toxic, and hypoallergenic, given pets’ active behavior and sensitive skin. Accurate monitoring requires sensor recalibration to pet-specific baselines, and integration with veterinary systems is important for meaningful use. Extended battery life with secure casing is critical to avoid accidental ingestion. Lastly, adapting human wearables for pets must account for animal-specific regulatory and ethical standards. This adoption also creates a symbiotic relationship where advancements in one domain benefit the other, enhancing device functionality and reliability across the board.

#### 4.1.2 Primary challenges

Among the various non-invasive and non-intrusive biomedical sensors and techniques, we focus on PPG and ECG. Both are popular and commonly used in human as well as veterinary medicine.

**Anatomical variety:** Wearable devices must accommodate substantial inter- and intra-species differences in breed, size, shape, behavior, skin type, and health needs. For example, dogs range from tiny Chihuahuas to large Great Danes, requiring adjustable straps and multiple size options. Different body shapes (e.g., slim Whippet vs. broad-chested Bulldog) and activity levels (e.g., high-energy Border Collie vs. low-activity Basset Hound) demand different design priorities. Certain breeds present specific challenges, brachycephalic dogs with respiratory issues or Dachshunds with spinal problems. Skin and fur characteristics also influence materials, such as the folded skin of Shar Peis, dense fur of Maine Coons, or hairless breeds like Sphynx cats.
**Recording concepts:** Fur lengths, densities, and skin properties [70] affect sensor performance. ECG electrodes require reliable skin contact through fur, potentially using needle or conductive rubber electrodes. Proper placement and secure attachment (special harnesses, elastic bands) help maintain signal quality while preventing discomfort or irritation [71].
**Technical design:** PPG sensors must be adapted for fur and skin variability, including wavelength and light-intensity optimization. Human PPG typically uses 660 nm (red) and 940 nm (IR), but wavelengths between 700–900 nm may be more effective for pets [72]. Experiments by Cugmas et al. show that placing sensors in less furry regions (ear, paw) improves reliability [70].
**Signal processing:** Breeds differ in physiological ranges, requiring calibration and tailored filtering to address higher noise and baseline wander. Dogs and cats exhibit higher heart and respiratory rates than humans [73, 74], and motion artifacts are more pronounced due to erratic movement, necessitating robust artifact-reduction algorithms.

#### 4.1.3 Data sharing and integrated platform

In addition to wearable devices, components such as data integration, analysis, decision-making, and secure data sharing remain underdeveloped. To date, no unified data platform exists that integrates health data from humans and animals (pets). Such a platform yields substantial advantages. By enabling data exchange between veterinary and human healthcare providers, it enhances clinical decision-making. Comprehensive health records across species lead to informed diagnoses and personalized treatments. Additionally, pet owners who actively manage and share their health data foster a collaborative healthcare environment where information flows efficiently. To ensure interoperability in pet-human wearable healthcare, we need to establish standards such as Fast Healthcare Interoperability Resources (FHIR). Beyond technical integration, ethical and regulatory considerations are essential for real-world deployment. Companion animals do not fall under human-focused data protection frameworks such as GDPR, yet responsible data governance, informed consent from owners, and transparent data-use policies remain critical for trust and adoption. Harmonized standards for interoperability, data formats, and device certification will be necessary to ensure safe, reliable, and scalable cross-species health data exchange.

### 4.2 Convergence towards Healthcare 5.0

Transitioning from a subject-to-device paradigm (hospital-centered), where technology is primarily confined to clinical settings, to a device-to-subject (patient-centered) approach has empowered individuals [75] and, increasingly, animals to participate actively in their health management. Wearable devices that continuously monitor environmental, behavioral, physiological, and psychological parameters have facilitated this shift [2]. The seamless integration of data from these devices into electronic health records (EHRs) has accelerated the move from Healthcare 2.0, characterized by data-driven decision making, towards a more proactive Healthcare 3.0. Other important technologies used in this revolution are big data analytics, block-chain technologies, artificial intelligence (AI), edge computing, and wearables powered by the Internet of Things (IoT) [76].

Precision prevention [77, 78] as a key component of Healthcare 4.0 relies on detailed personal and, now, even animal health information to tailor interventions. Wearable devices provide granular data on both human and animal health status, facilitate the identification of at-risk populations, and support preventive measures. By collecting data from humans and animals on a large scale, these devices contribute to public health surveillance and the early detection of emerging threats, including zoonotic diseases. The COVID-19 pandemic highlighted the critical role of wearable technology in tracking disease spread and informing public health interventions [79].

To realize the full potential of wearable devices in ODH, we also need to address ethical considerations. Establishing robust data governance frameworks and ensuring data protection are essential for building trust among users. We further need to develop standardized data formats and protocols to facilitate interoperable data exchange between different healthcare systems and devices across species. Techniques such as federated learning, differential privacy [80], secure multi-party computation [81], homomorphic encryption [82], and trusted execution environments [83] provide insights from private data while maintaining confidentiality. However, these techniques also face challenges such as communication overhead, system and data heterogeneity, and the need for robust security measures to prevent data leakage and model poisoning. By embracing the power of wearable technology and fostering collaboration between human and animal health sectors, we can create a Healthcare 5.0 future where diseases are anticipated, prevented, and managed more effectively for all living beings.

## 5 Conclusion

Integrating wearable devices into health monitoring enables unified human and pet health management within the ODH framework. Shared health metrics support early detection, chronic condition management, prevention of zoonotic diseases, and quality of life for both humans and animals. Re-purposing human wearables for pets reduces costs and accelerates adoption, promoting mutual benefit. A unified Healthcare 5.0 platform allows comprehensive data analysis for coordinated care, while standardized formats and secure data handling address critical interoperability challenges. Recognizing common environmental stressors further supports proactive, cross-species health strategies. Overall, cohesive integration of wearable technologies fosters healthier, more connected human–animal ecosystems. Most importantly, cross-species data integration creates a shared predictive foundation that enables earlier risk detection, more precise preventive interventions, and a new generation of anticipatory healthcare for both humans and companion animals.

## Data Availability

The original contributions presented in the study are included in the article/Supplementary Material, further inquiries can be directed to the corresponding author.
